# Evaluation of the new Chinese Disseminated Intravascular Coagulation Scoring System in critically ill patients: A multicenter prospective study

**DOI:** 10.1038/s41598-017-09190-5

**Published:** 2017-08-22

**Authors:** Yingying Wu, Lili Luo, Ting Niu, Yue Han, Ying Feng, Qiulan Ding, Ruibin Huang, Xiaohui Zhang, Jianming Feng, Ming Hou, Jun Peng, Yan Li, Yuhong Zhou, Lei Su, Linhua Yang, Zeping Zhou, Feng Xue, Jian Gu, Tienan Zhu, Xiaomin Wang, Jun Deng, Heng Mei, Yu Hu

**Affiliations:** 1 Institute of Hematology, Union Hospital, Tongji Medical College, Huazhong University of Science and Technology, Wuhan, P.R. China; 2 Department of Hematology, West China Hospital, Sichuan University, Sichuan, P.R. China; 3 Department of Hematology, First Affiliated Hospital of Soochow University, Suzhou, P.R. China; 4 Department of Hematology, Second Affiliated Hospital of Guangzhou Medical University, Guangzhou, P.R. China; 5 Department of Laboratory Medicine, Ruijin Hospital Affiliated to Shanghai Jiao Tong University School of Medicine, Shanghai, P.R. China; 6 Department of Hematology, First Affiliated Hospital of Nanchang University, Nanchang, P.R. China; 7 Department of Hematology, Peking University People’s Hospital, Beijing, P.R. China; 8 Department of Hematology, Qinghai Provincial People’s Hospital, Xining, P.R. China; 9 Department of Hematology, Qilu Hospital of Shandong University, Jinan, P.R. China; 10 Department of Hematology, First Hospital of China Medical University, Shenyang, P.R. China; 11 Department of Hematology, First Affiliated Hospital of Zhejiang Chinese Medical University, Hangzhou, P.R. China; 12 Department of Intensive Care Unit, General Hospital of Guangzhou Military Command, Guangzhou, P.R. China; 13 Department of Hematology, Second Hospital of Shanxi Medical University, Taiyuan, P.R. China; 14 Department of Hematology, Second Affiliated Hospital of Kunming Medical College, Kunming, P.R. China; 15 Institute of Hematology and Blood Diseases Hospital, Chinese Academy of Medical Sciences & Peking Union Medical College, Tianjin, P.R. China; 16 Department of Hematology, Clinical Medical College of Yangzhou University, Yangzhou, P.R. China; 17 Department of Hematology, Peking Union Medical College Hospital, Chinese Academy of Medical Sciences and Peking Union Medical College, Beijing, P.R. China; 18 Department of Hematology, People’s Hospital of Xinjiang Uygur Autonomous Region, Xinjiang Uygur Autonomous Region, P.R. China

## Abstract

Disseminated intravascular coagulation (DIC) is a common life-threatening complication in critically ill patients. The diagnostic scoring systems of DIC enable a more prompt and accurate diagnosis of DIC, such as the International Society on Thrombosis and Haemostasis (ISTH), the Japanese Association for Acute Medicine (JAAM) and the Japanese Ministry of Health and Welfare (JMHW). This study prospectively evaluated the newly proposed Chinese DIC Scoring System (CDSS), which was conducted at 18 centers in China during a one-year period. Receiver operating characteristic (ROC) curves showed that, for diagnosis of DIC and for prediction of the 28-day all-cause mortality, the CDSS had larger areas under the ROC curve (AUCs) than the ISTH and the JAAM in different groups. The CDSS also had larger AUC than the JMHW for the ISTH DIC in non-infectious diseases. All of the AUCs of the CDSS were greater than 0.8, accompanied with both high sensitivity and high specificity. Furthermore, the CDSS score was an independent predictor of mortality (odds ratio, 1.882; p < 0.001), and could reflect the illness severity (p < 0.001 for Spearman’s rank correlations with the scores of severity). In conclusion, the CDSS is worthy of promotion with a better diagnostic and prognostic value for DIC.

## Introduction

Disseminated intravascular coagulation (DIC) is a frequent and serious complication in critically ill patients with a high mortality. Prompt recognition of DIC is desirable for timely intervention and early treatment to improve the outcome of patients^1–3^.

In China, the guideline for the diagnosis of DIC was first proposed in 1986. In the last three decades, the guideline has been periodically updated according to its clinical application and the ever-increasing understanding of DIC. The latest version was established in 2012 by the *Consensus of Chinese Experts on Diagnosis and Treatment of DIC* ^4^. According to this consensus, a diagnosis of DIC is established when the patient has underlying diseases which may cause DIC, accompanied with clinical manifestations and (or) abnormal laboratory findings (Supplemental Table 1). The advancement in the diagnosis of DIC has greatly improved the management of DIC in China. However, limitations still exist in this consensus because it is a qualitative diagnostic standard for DIC. Not all the laboratory tests in this criteria have specific cut-off values for detecting abnormality, and the consensus does not assign quantifiable weights to individual laboratory and clinical parameters for DIC diagnosis. Therefore, the abnormal degree of certain laboratory findings and the contributions of each parameter to the diagnosis of DIC require a subjective judgement by clinicians based on their own clinical experience, thus inevitably leading to inconsistency in the conclusions of different clinicians. Furthermore, as a qualitative diagnostic standard, the consensus cannot be assessed repeatedly for dynamic monitoring of patients and evaluation of therapeutic effect. Therefore, a quantitative scoring system is much preferable to the consensus for the diagnosis of DIC.

Several scoring systems of DIC have been proposed, such as the Japanese Ministry of Health and Welfare (JMHW) DIC criteria^5^, the International Society on Thrombosis and Haemostasis (ISTH) overt DIC criteria^3^, and the Japanese Association for Acute Medicine (JAAM) DIC criteria^6, 7^, which are most commonly used in the world. Although all of them are demonstrated to be useful for the diagnosis of DIC, controversy still exists in the diagnostic performance and applicability of these criteria in different clinical settings^7–15^, thus making it confusing for clinicians to select an optimal scoring system in clinical practice.

For the sake of establishing a diagnostic scoring system for DIC which not only has both high sensitivity and high specificity in different clinical settings, but also is suited to the current clinical practice of China and even worth of promotion throughout the world, the Chinese DIC Scoring System (CDSS) was proposed in 2014. The diagnostic and prognostic value of the CDSS had been retrospectively validated in a single-center study previously^16^. This study was aimed at prospectively evaluating the CDSS criteria in various underlying diseases at multiple centers in China.

## Methods

The study protocol was approved by the ethics committee of Tongji Medical College, Huazhong University of Science and Technology. Written informed consent was obtained from the patients or the next of kin. The data collection was performed as a routine clinical workup without any interventions, and the data management and statistical analyses were processed anonymously. All the work was performed in accordance with relevant guidelines and regulations.

### Patients and selection criteria

753 patients were enrolled in this study conducted at 18 tertiary hospitals in China from June 2015 to July 2016. Patients were eligible when they had more than one of the following abnormal laboratory findings^12^: (1) platelet count <120 × 10^9^/L; (2) prolongation of prothrombin time (PT) >3 s; (3) fibrinogen level <1.0 g/L; 4) fibrin/fibrinogen degradation products (FDP) >10 mg/L; and 5) D-dimer >5 mg/L. Patients who met the following criteria were excluded: (1) <17 or >70 years of age; (2) liver cirrhosis classified as Child-Pugh grade C; (3) known clotting disorders, including anti-phospholipid syndrome, thrombotic thrombocytopenic purpura and heparin-induced thrombocytopenia. Evaluation of the CDSS in hematological malignancies was performed separately.

### Data sampling and Evaluation of Patients

Laboratory tests and clinical manifestations were collected when the patients were confirmed to be enrolled (day 1), as well as on day 2, 3 and 7 if possible. The scores of the CDSS DIC, the JMHW DIC, the ISTH overt DIC (ISTH DIC), and the JAAM DIC criteria were assessed simultaneously. Illness severity was evaluated according to the Acute Physiology and Chronic Health Evaluation (APACHE) II score^17^, while organ failure and inflammatory condition were assessed according to the Sequential Organ Failure Assessment (SOFA) score^18^ and the Systemic Inflammation Response Syndrome (SIRS) score^19^. All patients were followed up for the prognosis until the 28th day after enrolment. The outcomes of the patients were recorded, and the 28-day all-cause mortality both in hospital and after discharge was evaluated.

### Definitions

Diagnosis of DIC by each scoring system was established when the maximum score within the first three days of enrolment met the cut-off value of individual criteria. Table 1 shows the diagnostic algorithm of the CDSS, while the JMHW, the ISTH and the JAAM can be found elsewhere^10, 12, 19^. Clinical manifestations in the CDSS and the JMHW criteria were restricted to the signs and symptoms which could not be directly attributed to the primary diseases. Organ dysfunction was defined as having a SOFA score >2^18, 20^. D-dimer was used for the fibrin-related marker in the ISTH criteria. No increase, moderated increase and strong increase were defined as D-dimer levels of <0.5 mg/L (the upper limit of normal D-dimer level), 0.5–5 mg/L, and ≥5 mg/L (ten times the upper limit of normal) respectively.

**Table 1:** Chinese Disseminated Intravascular Coagulation (DIC) Scoring System (CDSS).

Item	Score
Underlying disease associated with DIC*	2
More than one of the following clinical manifestations independent of original diseases:	1
(1) Bleeding;	
(2) Organ dysfunction;	
(3) Microcirculatory disorder.	
Platelet count (×10^9^/L)	
<80	2
<100 and ≥80, or ≥50% decrease within 24 hours	1
≥100	0
Prolongation of prothrombin time (PT) and activated partial thromboplastin time (APTT) (s)	
Prolongation of PT ≥6	2
Prolongation of PT ≥3 and <6, or prolongation of APTT ≥10	1
Prolongation of PT <3 and prolongation of APTT <10	0
D-dimer (mg/L)	
≥9	3
≥5 and <9	2
<5	0
Fibrinogen (g/L)	
<1.0	1
≥1.0	0
Diagnosis of DIC	≥7

*If underlying disease is hematological malignancy: (1) No point for bleeding; (2) 1 point for platelet count of <50 × 109/L or ≥50% decrease within 24 hours; 3) Diagnosis of DIC is ≥6.

### Statistical analyses

Measurements are expressed as the mean ± standard deviation for continuous variables. Comparisons between two groups were made using Mann-Whitney’s U-test and the chi-square test, or Fisher’s exact test when necessary. Because there is no gold standard for the diagnosis of DIC, the JMHW DIC, the ISTH overt DIC and the JAAM DIC were used as the relative standard respectively for receiver operating characteristic (ROC) analyses to evaluate the diagnostic performance of the CDSS. ROC curves for the 28-day all-cause mortality were also constructed to compare the ability to predict mortality of the four scoring systems. The predictive ability of the CDSS for mortality was further verified by the stepwise logistic regression analysis (backward elimination based on the likelihood ratio), with outcome as the criterion variate, and age, gender and DIC score as the explanatory variates. The result was reported as odds ratio (OR) with 95% confidence interval (CI). Correlations of the CDSS score with the APACHE II score and the SOFA score were examined according to the Spearman’s rank correlation coefficient. The IBM SPSS 21.0 and MedCalc 15.6 for Windows software program was used for statistical calculations and analyses. For all reported results, p < 0.05 was considered to be statistically significant.

## Results

### Baseline characteristics of patients

During the 13-month study period, a total of 753 patients were enrolled, with underlying diseases including sepsis/severe infection (31.2%), trauma or surgery (21.5%), obstetric calamities (8.5%), vascular abnormalities (7.6%), acute pancreatitis (7.4%), solid tumors (7.2%), autoimmune diseases (5.2%), uremia/nephrotic syndrome (3.2%), severe hepatic failure (not Child-Pugh grade C) (2.5%), cardiogenic or hemorrhagic shock (1.5%), heat stroke (0.9%), severe toxic or immunological reactions (0.7%) and others^1, 2^.

**Figure 1:**
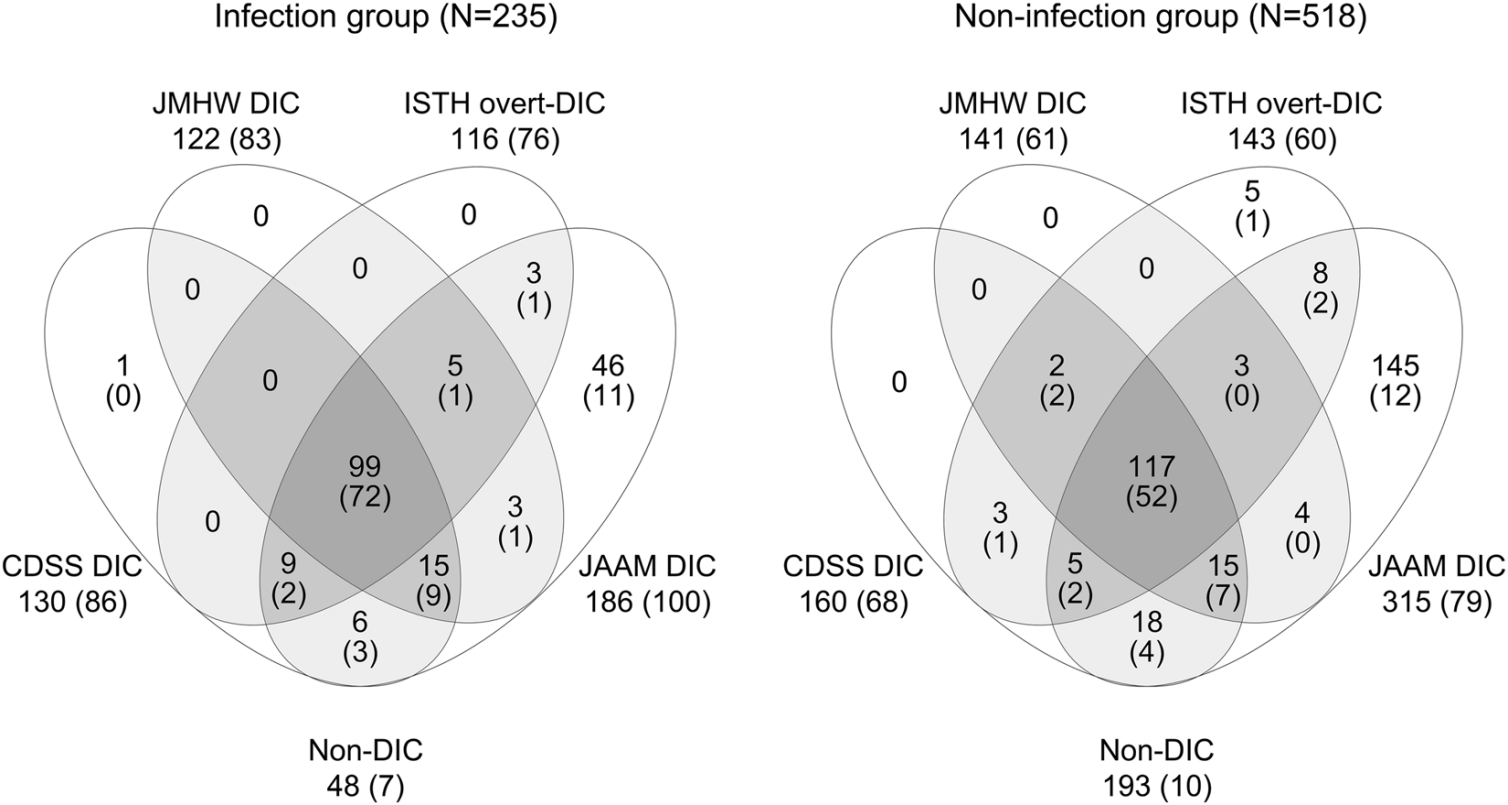
Relationships of the patients with disseminated intravascular coagulation (DIC) diagnosed by the four criteria. Left, comparisons in infection group; right, comparisons in non-infection group. CDSS, Chinese DIC Scoring System; JMHW, Japanese Ministry of Health and Welfare; ISTH, International Society on Thrombosis and Hemostasis; JAAM, Japanese Association for Acute Medicine; non-DIC, patients who didn’t meet the four criteria simultaneously. Numbers in parentheses are of nonsurvivors.

In the following analyses, the patients were divided into infection group with sepsis/severe infection and non-infection group with other underlying diseases. The prevalence of DIC within the first three days of enrolment by the CDSS, the JMHW, the ISTH and the JAAM were 55.3%, 51.9%, 49.4% and 79.1% (all p < 0.05 vs. CDSS) respectively in infection group, and 30.9%, 27.2%, 27.6% and 60.8% (all p < 0.05 vs. CDSS) respectively in non-infection group. The relationships of the DIC diagnosed by the four criteria are shown in Fig. 1. Baseline characteristics and the 28-day all-cause mortality of the patients are presented in Table 2. No patients died of accidents during the period of follow-up. Intriguingly, the prevalence of DIC and the mortality of DIC by each set of diagnostic criteria were significantly higher in infection group than in non-infection group.

**Table 2:** Baseline characteristics of the patients.

	Infection group		Non-infection group	
	CDSS non-DIC	CDSS DIC	CDSS non-DIC	CDSS DIC
Age (years)	50.3 ± 14.8	52.0 ± 13.0	48.2 ± 13.6	47.0 ± 15.4
Male:female	57:48	79:51	194:164	84:76
APACHE II score	13.8 ± 8.7	22.6 ± 12.0^a^	10.8 ± 5.5^b^	19.7 ± 10.4^a^
SOFA score	4.7 ± 4.3	10.3 ± 5.2^a^	3.0 ± 2.7^b^	8.8 ± 5.0^a,b^
SIRS score	2.2 ± 1.2	2.9 ± 1.0^a^	1.6 ± 1.2^b^	2.4 ± 1.1^a,b^
28-day mortality	21/105, 20.0%	86/130, 66.2%^a^	25/358, 7.0%^b^	68/160, 42.5%^a,b^

DIC, disseminated intravascular coagulation; CDSS, Chinese DIC Scoring System; JMHW, Japanese Ministry of Health and Welfare; ISTH, International Society on Thrombosis and Haemostasis; JAAM, Japanese Association for Acute Medicine; APACHE, Acute Physiology and Chronic Health Evaluation; SOFA, Sequential Organ Failure Assessment; SIRS, systemic inflammation response syndrome. ^a^p < 0.05 between CDSS DIC and CDSS non-DIC; ^b^p < 0.05 between infection and non-infection.

### Diagnostic performance of the CDSS

ROC curves for the relative standard were constructed respectively (Table 3). The results showed that the CDSS had higher sensitivity than the ISTH for the JMHW DIC and the JAAM DIC (both p < 0.05 in the two groups). The CDSS also had higher sensitivity than the JMHW for the ISTH DIC (p < 0.05 in infection group) and the JAAM DIC (p < 0.05 in non-infection group). The areas under the ROC curve (AUCs) of the CDSS for the DIC diagnosed by the relative standards were all greater than 0.87.

**Table 3:** Receiver operating characteristic (ROC) analyses for the relative standard

	JMHW DIC			ISTH overt-DIC			JAAM DIC		
	CDSS	ISTH	JAAM	CDSS	JMHW	JAAM	CDSS	JMHW	ISTH
Infection group									
Sensitivity (%)	93.4	85.2^a^	100	93.1	89.7^a^	100	69.4	65.6	62.4^a^
Specificity (%)	85.8	89.4	43.4^a,b^	81.5	84.9	41.2^a,c^	98.0	100	100
AUC^d^	0.957	0.927^a^	0.932	0.944	0.946^a^	0.874^a,c^	0.926	0.945	0.898^c^
Non-infection group									
Sensitivity (%)	95.0	86.5^a^	98.6^b^	88.8	85.3	93.0^c^	49.2	44.1^a^	42.2^a^
Specificity (%)	93.1	94.4	53.3^a,b^	91.2	94.9^a^	51.5^a,c^	97.5	99.0	95.1^c^
AUC^d^	0.985	0.965^a^	0.934^a,b^	0.968	0.967	0.880^a,c^	0.875	0.918^a^	0.812^a,c^

CDSS, Chinese DIC Scoring System; JMHW, Japanese Ministry of Health and Welfare; ISTH, International Society on Thrombosis and Haemostasis; JAAM, Japanese Association for Acute Medicine; AUC, area under the ROC curve. ^a^p < 0.05 vs. CDSS; ^b^p < 0.05 vs. ISTH; ^c^p < 0.05 vs. JMHW; ^d^p < 0.001 for each AUC.

### Prognostic value of the CDSS

ROC analyses for the 28-day all-cause mortality were conducted (Table 4). The results showed that the CDSS exhibited higher sensitivity for mortality than the JMHW (p < 0.05 in infection group) and the ISTH (p < 0.05 in non-infection group), while the JAAM had the highest sensitivity and the ISTH had the highest specificity both in the two groups. The AUCs of the CDSS for mortality were smaller than that of the JMHW, but the differences were not significant either in infection group (0.808 vs. 0.827, p > 0.05) or in non-infection group (0.804 vs. 0.822, p > 0.05).

**Table 4:** Receiver operating characteristic (ROC) analyses for the 28-day all-cause mortality

	CDSS	JMHW	ISTH	JAAM
Infection group				
Sensitivity (%)	80.4	77.6	71.0^a^	93.5^a,b,c^
Specificity (%)	65.6	69.5	68.7	32.8^a,b,c^
AUC^d^	0.808	0.827	0.764^a,b^	0.768^b^
Non-infection group				
Sensitivity (%)	73.1	65.6^a^	64.5	84.9^a,b,c^
Specificity (%)	78.4	81.2^a^	80.5	44.5^a,b,c^
AUC^d^	0.804	0.822	0.793	0.760^a,b^

CDSS, Chinese Disseminated Intravascular Coagulation Scoring System; JMHW, Japanese Ministry of Health and Welfare; ISTH, International Society on Thrombosis and Haemostasis; JAAM, Japanese Association for Acute Medicine; AUC, area under the ROC curve. ^a^p < 0.05 vs. CDSS; ^b^p < 0.05 vs. JMHW; ^c^p < 0.05 vs. ISTH; ^d^p < 0.001 for each AUC.

The patients with the CDSS DIC had significantly higher 28-day mortality rate than those without the CDSS DIC both in the two groups (Table 2). For the patients diagnosed with CDSS DIC within the first three days, those who still met the DIC criteria on day 7 had significantly higher 28-day mortality than those who did not (24/45, 53.3% vs. 7/45, 15.6%; p < 0.001). For the patients without CDSS DIC within the first three days, those with new-onset DIC on day 7 had significantly higher mortality than those who never developed DIC during the follow-up period (5/12, 41.7% vs. 4/106, 3.8%; p < 0.001).

Furthermore, the logistic regression analysis for the 28-day mortality further demonstrated a strong correlation between an increasing CDSS DIC score and mortality. For each one-point increment in the CDSS DIC score, the OR for mortality was 1.882 (CI 1.699–2.083, p < 0.001).

### Reflection of illness severity by the CDSS

**Figure 2:**
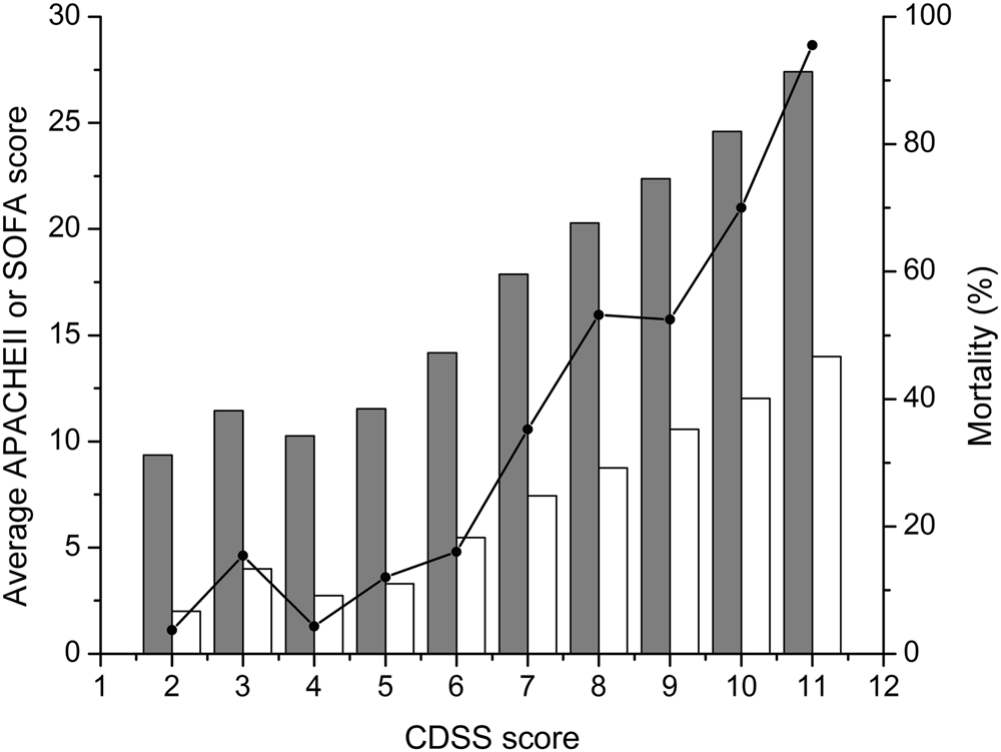
Corellations between the CDSS score and the APACHE II score, the SOFA score as well as the 28-day all-cause mortality. With the increase of the Chinese Disseminated Intravascular Coagulation Scoring System (CDSS) score on the day of diagnosis, average Acute Physiology and Chronic Health Evaluation (APACHE) II score (white bars), average Sequential Organ Failure Assesssment (SOFA) score (gray bars) and the 28-day all-cause mortality (line) of the patients increased simultaneously.

The patients with the CDSS DIC had significantly higher APACHE II score and higher SOFA score on the day of diagnosis than those without the CDSS DIC both in infection group and in non-infection group (Table 2). With the increase of the CDSS DIC score, the APACHE II score and the SOFA score of the patients increased simultaneously (Fig. 2). The Spearman’s rank correlation coefficients of the CDSS DIC score with the APACHE II score and the SOFA score were 0.488 and 0.643 (both p < 0.001) respectively.

## Discussion

The JMHW, the ISTH and the JAAM criteria are the most widely used scoring systems of DIC in the world currently. Although all of these criteria have been demonstrated to be useful for the diagnosis of DIC, the applicability and diagnostic performance of these criteria in different clinical settings remains controversial. In previous studies, both the JMHW and the ISTH had moderate sensitivity and specificity for the diagnosis of DIC, while the JAAM had extremely high sensitivity but relatively low specificity. Therefore, the JAAM may be appropriate for screening of early-phase DIC while the JMHW and the ISTH can be used for confirming diagnosis of DIC. Furthermore, the JAAM was created primarily for infectious and septic DIC, while the JMHW uses separate diagnostic algorithms for DIC in hematological malignancies and in other diseases. Therefore, it is always complicated and confusing for clinicians to select an optimal scoring system in different conditions in clinical practice. The CDSS is therefore proposed for the sake of establishing an integral scoring system of DIC which not only has both high sensitivity and high specificity but also applies to different clinical settings.

Compared with the established scoring systems, the CDSS has the following features. 1) The CDSS takes the underlying disorders as one of its integral components instead of a *conditio sine qua non* in DIC diagnosis, thus increasing its sensitivity in patients who are suspected of DIC but do not exhibit obvious disorders related to DIC. 2) The CDSS emphasizes the role of clinical manifestations in DIC diagnosis, in consideration of that they are important clues for clinicians and have a significant impact on the prognosis of DIC patients. 3) The CDSS includes the prolongation of activated partial thromboplastin time (APTT) in addition to the prolongation of PT, which increases its sensitivity in patients with prolonged APTT but not prolonged PT. 4) D-dimer is used as the fibrin-related marker in the CDSS, because D-dimer rather than FDP can discriminate between degradation products of cross-linked fibrin and fibrinogen^21^, and the former is demonstrated to have larger AUC for the diagnosis of DIC^16^. 5) The CDSS includes the dynamic decrease of platelet count in addition to the absolute platelet count, thus increasing its sensitivity in patients who develop DIC but still have platelet count remaining in normal range or have received platelet transfusion. 6) Because thrombocytopenia and hemorrhage are common complications in patients with hematological malignancies, the CDSS removes hemorrhage from the item of clinical manifestations, and assigns lower cut-off value and smaller power to the platelet count for DIC in hematological malignancies.

Good diagnostic criteria should meet three conditions: 1) be readily available and easy to use for each clinician; 2) have good diagnostic performance with appropriate sensitivity and specificity; and 3) display prognostic value for monitoring and management of patients^22^. The CDSS adopts the markers of coagulation and fibrinolysis (Table 1) that are routine tests and readily available at all hospitals. Clinical manifestations are also included in the scoring system, the recognition of which may not be challenging for clinicians. Therefore, the CDSS criteria can be easy to grasp and implement.

Systemic infection or sepsis are among the most common causes of DIC, and may be complicated by DIC in 30–50% of cases^1, 2^. The pathogenesis of infection-induced DIC is characterized by the interplay between inflammation and coagulation, as well as the hypofibrinolysis which results in enhanced intravascular fibrin deposition and high incidence of MODS. The JAAM was proposed to focus on infection-related DIC, which includes SIRS criteria in its algorithm^6, 13–15^. By comparing the CDSS with the other scoring systems in infection group and in non-infection group separately, we were able to assess whether the diagnostic performance of these criteria varied in different situations. Actually, it would be better to evaluate these criteria in different underlying diseases respectively if the sample size were large enough.

In our study, sepsis/severe infection accounted for the largest proportion (31.2%) among different underlying diseases. Both in infection group and in non-infection group, the DIC diagnostic rate of the CDSS was higher than that of the JMHW and the ISTH, but lower than that of the JAAM. The ROC analyses for the relative standards showed that, the CDSS always exhibited higher sensitivity than the JMHW and the ISTH, and higher specificity than the JAAM. Although the specificity of the CDSS was lower than that of the JMHW and the ISTH, its AUCs were larger than those of the ISTH and the JAAM. The CDSS also had larger AUC than the JMHW for the ISTH DIC in non-infection group, and all the AUCs of the CDSS were greater than 0.87 for the DIC diagnosed by the relative standards. In summary, the CDSS exhibited both high sensitivity and high specificity for the diagnosis of DIC, thus avoiding both missed diagnosis and misdiagnosis at the same time.

ROC analyses for the 28-day all-cause mortality showed that the CDSS also had higher sensitivity for mortality than the JMHW and the ISTH. Though the AUCs of the CDSS for mortality were smaller than those of the JMHW, the differences were not significant and all the AUCs of the two criteria were greater than 0.8 in the subgroups of patients. The logistic regression analyses further demonstrated that the CDSS DIC score was an independent predictor of mortality. In addition, the good correlation of the CDSS DIC score with the APACHE II score and the SOFA score indicated that the CDSS DIC score could also reflect the illness severity of patients.

It should be noted that these conventional markers used in these scoring systems are still of limited value in the accurate recognition of DIC at early phase^19, 21^. Therefore, more and more attention has been paid to the diagnostic value of special molecular markers which play pivotal roles in the pathogenesis of DIC, including thrombin-antithrombin complex, soluble fibrin, plasmin-plasmin inhibitor complex and plasminogen activator inhibitor-1. New scoring systems adopted these markers were demonstrated to be highly sensitive and specific for diagnosis of early-phase DIC and for prediction of mortality^23, 24^. Therefore, it is worthwhile to further investigate their utility in our scoring system, and the study of which is being carried out in another multicenter prospective study currently.

## Conclusions

This multicenter study prospectively evaluated the diagnostic performance and prognostic value of the newly established Chinese DIC Scoring System. Compared with the JMHW, the ISTH and the JAAM, the CDSS criteria exhibited good diagnostic performance for DIC, which gave consideration to both sensitivity and specificity in different underlying diseases. Furthermore, the CDSS DIC score also had a good prognostic value, which could not only predict the outcome but also reflect the illness severity of patients. The wide application of CDSS will greatly improve the unified and standardized diagnosis of DIC in China.

## Electronic supplementary material

Supplementary Information
