# Supplementary material for: Evaluation of the new Chinese Disseminated Intravascular Coagulation Scoring System in critically ill patients: A multicenter prospective study
Source: Sci Rep. 2017 Aug 22;7:9057. doi: 10.1038/s41598-017-09190-5 (PMC5567287; doi:10.1038/s41598-017-09190-5)
Supplement: Supplementary file 1 — Supplementary Information [file 41598_2017_9190_MOESM1_ESM.doc]

Supplemental Table 1 Consensus of Chinese Experts on Diagnosis of Disseminated Intravascular Coagulation*

| 1. Presence of underlying diseases 2. Having more than one clinical manifestation which cannot be explained by original diseases: 3. Tendency of multiple hemorrhage; 4. Microcirculatory failure or shock; 5. Symptoms or signs of multiple microembolism. 6. Having more than three of the following abnormal laboratory tests: 7. Platelet count <100 ×10^9^/L, or has a sustained declining; 8. Fibrinogen <1.5g/L, or has a sustained declining; 9. Fibrin/fibrinogen degradation products >20mg/L, or D-dimer increases; 10. Prolongation of prothrombin time >3s, or prolongation of activated partial thromboplastin time >10s. |
| --- |

*The original edition is in Chinese.
